# Pakistani Student Nurses’ perceptions of their hospital’s health professionals’ attitudes and suggested ways to improve patient care – An untainted view

**DOI:** 10.12669/pjms.36.7.3110

**Published:** 2020

**Authors:** Sachal Aqeel Safdar, Humaira Zafar, Jawwad Ahmad, Rashid Qayyum, Sajid Naseem, Chaudhry Aqeel Safdar

**Affiliations:** 1 Dr. Sachal Aqeel Safdar, MBBS. Shifa International Hospital, Islamabad - Pakistan; 2 Dr. Humaira Zafar, MBBS. Fazaia Medical College/ PAF Hospital & Air University, Islamabad, Pakistan; 3 Jawwad Ahmad, FCPS (Ophth), FCPS (Vireo-retinal) Associate Professor, Fazaia Medical College/ PAF Hospital & Air University, Islamabad, Pakistan; 4 Rashid Qayyum, FCPS (Psych) Associate Professor, Fazaia Medical College/ PAF Hospital & Air University, Islamabad, Pakistan; 5 Sajid Nasim, FRCP (UK) Assistant Professor, Fazaia Medical College/ PAF Hospital & Air University, Islamabad, Pakistan; 6 Prof. Chaudhry Aqeel Safdar, FRCSEd, MCPS-HPE, MSc Fazaia Medical College/ PAF Hospital & Air University, Islamabad, Pakistan

**Keywords:** Nurses, Education, Perceptions, Feedback

## Abstract

**Background & Objectives:**

Feedback brings a fresh perspective and improvement in any organization. Health professionals (HPs) lose insight of the gaps in medical care. The views of student nurses can help improve systems. The objective of this study was to assess the views of our student nurses and how they perceive the way the doctors and HPs work in our hospital and comment on training, attitudes, care pathways, teamwork, and what needed to be improved.

**Methods:**

A proforma based qualitative study was carried out at the Nurses’ Training Centre of PAF Hospital and Fazaia Medical College, Islamabad, from January to March 2020. After approval, a semi-structured proforma with open and closed ended questions was administered, in English and Urdu. The results were analyzed by comparative numbers and percentages for each question and descriptive responses were grouped in recurring themes and analyzed for content and their constructive value.

**Results:**

Out of 85 nursing cadets, the proforma could be administered to 61(M=38(62.3%) and F=23(37.7%). Most were FSc with 26% graduates. Majority of the female students’ main reason for joining was to serve humanity, unlike most males. According to gender many responses were interestingly different. Majority of females thought male doctors were better (86%). Only 36% said the doctors were sincere in care of patients. Most thought that we needed to improve patient counseling. Most thought the seniors treated them unfairly, but bullying was negligible. They wanted the senior HPs to improve their attitudes and ensure adequate equipment in the wards. They were worried about personal security from patients and relatives. Dedicated mental health services to deal with stress of witnessing every day misery and death was suggested.

**Conclusions:**

Doctors need to improve their counseling skills and should talk more to the patients and their relatives. They should acknowledge the nursing students and improve teamwork. Belittling them in front of others harms their self-efficacy. Simple corrections like punctuality, ownership of their patients and improvement of equipment and systems can improve patient care.

## INTRODUCTION

One of the greatest strengths of Nurses training all over the world is their direct involvement in the care of the patients almost from the very first day.^1^ They have lectures, usually, in the morning and then perform supervised duties in the wards, doing regular shifts, including night duties. This is very different from the medical students training who spend nearly half of their training hours in the class rooms or labs. It also gives the nurses a unique, untainted perspective about the way the patients are treated in their hospital and about those who provide this care. They have not been exposed to the somewhat cynical world of medical profession and their “war (d)-hardened” seniors with their lessening empathy.^2^

As many of us have observed, and has been proven, that the newly inducted health professionals are motivated, empathetic and altruistic. But over the years they lose these essential qualities.^3^ This loss of empathy in health professionals (HPs) and medical students occurs in the clinical years; more so during house jobs and training years. Many reasons have been given and ways to improve proposed.^4^ These HPs in training are silent and often invisible soldiers of medical profession and are in a unique position to sense and observe the way we work and what needs to be improved.^5^

Feedback from lay people or juniors often brings a fresh perspective and improvement. These feedbacks have content, construct and predictive validity.^6^ After years of working in the harsh hospital environments and being “battered” by the physical and psychological salvos, the ‘touch’ withers with loss of empathy.^7^ They are patients’ advocates, and hence should be encouraged to contribute in the care, a system practiced in UK, although not so much in German system.^8^ We sometimes get entrenched in our own set archaic ways and views from these new nurse students may provide a fresh insight of our training and care shortcomings.^9^ It may be useful to harness the views of these early years of the new nurses, who perhaps know more about the way doctors and other HPs work and deal with their patients, thus improving our patient care and the way we deal with these junior-most, but key players of our teams.^10^

Based on the above surmises, the objective of this study was to administer a survey to our student nurses and assess their views about how they perceive the way the doctors and senior HPs work in our hospital. The emphasis was to comment on attitudes of HPs regarding training, care pathways, teamwork, and what needed to be improved.

## METHODS

This was a proforma based qualitative exploratory study conducted at the Nurses’ Training Centre of Pakistan Air Force (PAF) Hospital and Fazaia Medical College, Islamabad, from January to March 2020. Approval from Institutional Review Board (IRB/01/3/07/2020) was obtained. A semi-structured proforma with simple open and closed ended questions was administered. The questions were both in English and Urdu, and replies could also be in both languages (Annexure 1). It was anonymously filled and consent taken.

The first section of the proforma noted the demographics and education with the basic baseline questions to provide a general background of each cadet. This helped to provide a better understanding of this cohort, and hence correlate with their perceptions. Then there were sections about their views and perceptions regarding: 1, the doctors they worked with; 2, the senior nurses and administrative staff, commenting on their attitudes and what needed to be improved; 3, what needed to be improved in the hospitals and patient care; 4; lastly, a section for free expression of views (Annexure 1).

The results were analyzed by finding percentages and frequencies for numerical variables, or as frequency or percentages for categorical variables for each closed question and descriptive responses of open ended questions were grouped in recurring themes and analyzed for content and their constructive value. They were also cross-analyzed for correlation with gender and other demographic information provided, using Microsoft Excel.

Frivolous responses and personal problems were identified but not included in the evaluation reports. Although they were shared with the tutors to determine their nature and address appropriately, ensuring that their genuine grievances are heard.^11^

## RESULTS

At the time of study, there were 85 nursing cadets of PAF Medical Services enrolled. The proforma could only be administered to 61 of them, the rest being on temporary duties or leave. The sex ratio was M=38(62.3%) and F=23(37.7%). A summary of their demographics and basic data are given in Table-I. Some of them provide interesting facts, which may give insight into their backgrounds and responses.

**Table-I:** Demographics of the survey population of the responding nursing students.

Total = 61	Males 38 (62.3%)		Females 23 (37.7%)	

Age	Average age = 21.5 years (Range 21 to 25 years)			
Qualification	Matric 6%	FSc 68%	BSc/ BA 26%	MSc/ MA 0%
Division / Grade	First 90%	Second 10%	Third 0%	
Year of Training	One 13%	Two 34%	Three 53%	
Province & District OF Domicile	Punjab 78% (Max: Chakwal & Attock)	KPK 16% (Karak & Lakki Marwat)	AJK 3%	Baluchistan 3% Sind 0%
Father’s Profession	Govt Servants 40%	Armed Forces Rtd 30%	Farmers 3%	Others: 27% Business, shopkeeper, Unemployed
Total Siblings	Average 4.6, Range 1 to 9, Max having between 4 and 6 siblings			

At the end of the Q 3 (what do the doctors of our hospital need to improve), Q 4 (what do they think about their own nursing seniors) and Q 6 (Anything else you want to say) it was encouraged to give free opinions. The main themes, answers and the percentages are given in Table-II. The main themes and top answers were:

- Why you came to nursing? (serve, honorable profession, smart uniform)
- Views about doctors? (Consultants best, Males better-opinion of female students and vice versa, house officers have best attitude)
- Doctors need to improve? (Talk to patients, be polite, nice to juniors, appreciate us)
- About your senior nurses? (Treat us badly, shout at us, some nice, don’t work themselves, unfair in duties, don’t teach)
- How to improve patient care? (Competent and polite doctors, improve facilities, more equipment, better ward care)
- Free opinions and answers to open ended questions are summarised in Table-III.

**Table-II:** Answers to the survey questions.

Q 1. Why you came into nursing	Serve humanity M- 50% F- 72%	Like smart uniform M- 33% F- 83%	For bright future M-25% F-55%	To escape home M- 0% F- 0%	Honorable profession M- 58% F- 55%	Good salary M.17% F-39%	Support family M- 58% F- 50%	Better marriage proposal M- 0% F- 22%	See the world M-17% F-22%	
Q 2. View about the Drs in hospital	Who are Best Drs HO 18% Tr 13% Con 69%	Who is better Dr, M or F? M Std= M 37% F 63% F Std= M 86% F 14%	F Std= HO 42% Tr 5% Con 53%	Best attitude to pts? HO 28% Tr 17% Con 55%	Drs sincere in their jobs? All 7% Most 34% Some 21% Few 36% None 2%	Drs honest to pts? HO 10% Tr 9% Con 81%	Drs good at their job Senior 12% Junior 16% Both 72%	Most punctual HO 19% Tr 9% Con 30% None 42%	Depts with best Drs 1.Paeds 2. Med 3. Surg 4. Cardi	
Q 3. Points to improve Drs	Talking to pts needs improving Yes 74%	Need to be polite Yes 63%	To be nice to nurses Yes M 58%, F 94%	Appreci-ate us more Yes 86%	Should do rounds on time Yes 70%	Dress up better Yes 32%	Male drs more improve Yes M 72% F 22%	Lady drs more improve Yes M 25% F 83%	Need better manners Yes 46%	Comments See below
Q 4. Views about your own senior nurses	Treat us badly Yes 73%	Shout without reason Yes M 0% F 55%	Some are good Yes 70%	Don’t Teach us Yes M 17% F 45%	Not fair for duty rosters Yes M 33% F 61%	Don’t work themselves Yes M 67% F 94%	Bully & swear Yes M 17% F 28%	Better, Males or females M 22% F 78%	Are helpful Yes 50%	Comments (see below)
Q 5. How to improve hospital & pt care	Get better Drs Yes 62%	More Drs Yes 22%	Reduce pts Yes 39%	Poor facilities / food etc Yes 78%	Better attitude of nurses Yes 67%	Free treatment Yes 45%	Ward care improvement Yes 71%	Emerg improvement Yes 31%	Overall care Improvement Yes 92%	More equipment Yes 72%

(M=Male Nursing students, F=Female Nursing students, Dr=Doctors, HO=House officers, Tr= Trainees, pts=patients; there are multiple responses for some questions)

The nursing cadets also mentioned some administrative problems related to the service. These were noted and conveyed to the senior administrators separately. They included points like: excuse of non-clinical duties, separate rooms for admitted staff in the hospital, and some others.

## DISCUSSION

The first section of the proforma explores the demographics of our cohort of the newer nurses, which has been found to be useful^12^ and presented some interesting information (Table-I). It is crucial to inspect the socio-demographics which can affect the validity of any survey.^13^ These were all uniformed nursing students of the PAF nursing services. In our study, 68% had completed their higher secondary school certificate (FSc), with 26% graduates and only 6% were matric (O-levels equivalent), while a decade ago in a study from Karachi had 78% of their nursing students with matric and only 20% FA/FSc, while only 2% were graduates^14^; 90% of our nurses had achieved first division in their exams, many missing entry into a medical school by only a few marks. Hence the intellectual importance of the responses to the survey questions.^15^ Most of them were from Punjab and KPK, the northern provinces of Pakistan, one from Baluchistan and none from Sindh. This was expected as those from the southern provinces would have gone to the Karachi institutes. Fathers of most of them (70%) were government servants or from armed forces, hence their liking for joining the PAF. They came from families with average number of siblings at 4.6, medium sized by our country’s average, with girls coming from bigger families. This supports the surmise that they would be able to support the family income. Another reason could be to secure a better marital proposal. In India, which is one of the biggest suppliers of trained nurses to the Middle and Far East, this has been quoted as an important factor for joining the nursing profession.^16^

The Question 1 in the survey asked why they wanted to join nursing in the PAF (Table-II). Altruistic motives of serving the humanity were stronger in the females (M vs F, 50% vs 72%). Forces uniform has always attracted young people, and 82% of the girls considered this to be one of the reasons for making this decision. Many boys and girls came from the village backgrounds and joining PAF is a good reason to escape the village life, but in our survey none of the boys or girls quoted this to be a reason (0%). Obviously with more and varied job opportunities available, coming to nursing was a considered choice. A change from previous studies.^16^

Question 2 was important and asked the views of the students regarding the doctors, with whom they worked. Overall they thought the Consultants were best ‘doctors’ (87%), compared to House officers-HOs 23% and Trainees-TRs 17%. This can be expected as the expertise of Consultants was an obvious choice for being selected. About 86% of female students thought that male doctors were better at their job, as compared to only 37% of male students. This gender bias which has been observed previously in other studies as well.^17^ However, only 36% thought that the doctors were sincere in their job, a disconcerting finding. Another important question was about the attitudes in dealing with the students themselves. In spite of the fact that the consultants were considered the best doctors, ***the HOs had the best attitude and working relationship with nurses***. The consultants ignored them, and this made the teamwork less than optimum. ***Consultants, as leaders, have to acknowledge the student nurses and make them a useful part of the teams in the care of the patients, which has been reported***.^18^ Another aspect pointed out was the punctuality, which fared very poorly, trainees and HOs being the least punctual. This is something which needs to be inculcated early into the young doctors, as previously shown.^19^ We also asked which departments were the best and in the order of merit, they were Paediatrics, Medicine, Surgery and Cardiology. Other major departments like Gynaecology, ICUs, Theatres, ER and diagnostics need to reflect on the reasons for this and this was conveyed.

When asked in Question 3, what the doctors need to improve; ***an overwhelming majority wanted the doctors to improve the way they talk to the patients, and to be polite***. This has been pointed out by so many studies which emphasize the need for empathetic communication skills. Mindfulness-based empathy training (MBET) leads to an increase in empathy.^20^ ***Punctuality*** was again found to be lacking, which can perhaps be improved by better role modeling and better institutional culture.^19^ ***Again the female students thought that female doctors need more improvement***. They also thought that ***doctors should be nice to student nurses in the wards and 86% wanted to be appreciated for the work they do***, which helps in team building, motivation and self-efficacy of the learners.^15^, ^21^

Question 4 addressed the attitudes of the senior nurses, tutors and managers. Majority thought that they treat them badly (73%) and unfairly (61%). During the feedback bullying was another direct question which was asked. Bullying is a critical issue, since it is linked with nurse attrition and eventually to their morale.^22^,^23^ Bullying also negatively affects victims’ social relationships inside and outside the institution.^24^ ***As a pleasant surprise only a very small number mentioned being subjected to bullying***. Although bullying and abuse has been reported.^11^ This has not been proven by this study, a credit to the institutional culture. Unstructured and one to one teaching was also found to be lacking. A good learning environment is dependent on engagement and collaboration between preceptors and academic nurse teachers.^15^,^25^ To produce a relaxed, empathetic, competent and confident HPs the institute needs to provide congenial and friendly educational environment.^26^ Work motivation among nurses is a crucial tool to manage nurses’ shortage, retention, migration and work leave.^27^

Question 5 was important to know, holistically, about the important aspects of improving the hospital care. ***Almost all (92%) thought the patient care needs improvement***. We expected that having more doctors and fewer patients would receive a yes vote. However, only 22% and 39% thought this was necessary. Most wanted doctors to do better (62%), better nursing attitudes (67%), more equipment (72%) and improving in-patient care systems (71%). This was a most insightful assessment from such a young group and reported from other studies.^28^

The last part of the survey asked for free comments (Table-III). One theme was ***to give any harsh feedback separately and not in front of the patients. It belittled them*.** This decreases confidence; better techniques can be used. The ‘feedback sandwich technique’ provided at the bedside are most useful to the student’s learning.^28^ ***The second important theme was that doctors lack in the way they communicate with the patients and their relatives***. The teams discuss the disease at length with each other but do not involve the patients and their carers in the discussions. They also need to care for the private and public patients equally, and irrespective of the status; a simple observation which senior doctors need to reflect on.^29^

**Table-III:** Summary of free comments in response to Q 3, Q 4 and Q 6 - main themes.

Q 3 (what do the doctors of our hospital need to improve)	Q 4 (what do they think about their own nursing seniors)	Q 6 (Anything else you want to say to improve hospital working)
*Male students:* 1. Not blame us all the time 2. Give respect to us, so that patients respect us too 3. If there are mistakes tell them separately and not in front of patients. 4. Show more empathy to patients. 5. Should not treat patients harshly. *Female students:* 1. A few doctors treat private and public patients differently. 2. Doctors discuss the disease and care plan with each other but not explain to the patients or relatives; counseling not adequate. 3. Be nice to patient’s attendants also. 4. Give equal care and not be biased. 5. There should be better communications with the patients.	*Male Students:* 1. Seniors worry more about discipline than actual nursing. 2. Seniors are knowledgeable but don’t teach much. 3. Seniors don’t encourage us and criticizing only 4. Senior staff need refresher courses and CPD. 5. Not use cadets for personal jobs and chores. 6. No favouritism. *ALL THESE ARE VERY VALID POINTS* *Female students:* 1. Don’t appreciate our good work at all. 2. Some treat us badly, as they were treated badly, themselves. 3. Improve their work ethics. 4. Some are kind, some less, and some outright rude. 5. Don’t bully but discouraging. 6. Improve atmosphere for learning. 7. Seniors direct their anger and frustrations at us. 8. Some male seniors enjoy telling us off.	*Male & Female Students:* *Patient Related:* 1. Patients coming after working hours misuse the service; hoard medicines. 2. Pts and their relative should also learn to behave… not only doctors. *Doctors Related:* 1. Need more doctors and nurses. 2. On OT days, somebody in the OPD also, and do rounds on time. 3. Specialists should come to OPD on time. 4. Pts should know who is their treating doctor and he should be responsible. 5. PGs behaviour needs improvement. They prescribe treatment without examining. 6. Avoid unnecessary admissions & not practice DEFENSIVE MEDICINE. 7. Too many referrals to Specialists. *Nursing Related:* 1. Improve physical and mental health of the staff. NO PSYCHOLOGICAL SUPPORT. 2. Modernize nursing curriculum. 3. Rotations in all wards 4. Staff should know their roles specifically *Hospital Related:* 1. Suggestion boxes in each ward 2. Crockery and the way food is served needs improvement. 3. More drinking water outlets. 4. Better administration 5. LOT OF DOCUMENTATION AND COMPUTER ENTRIES. THERE IS LESS TIME FOR ACTUAL NURSING 6. Wards in UNIT I need maintenance. 7. New & modern beds

The section 6 of the survey was an invitation to give free comments. It was mentioned that, not only the HPs of the hospital, but the ***patients and their relatives*** also need to follow a code of ethics. This should be implemented and unruly behaviour should be checked immediately to prevent from escalating.^30^ Some other comments about ***doctors’ practices*** were also insightful. In some wards the rounds were carried out by different doctors every day. This often caused confusion about their main doctor. Regarding the ***nursing care***, clear defining of roles of each member of the ward team was pointed out. This effects job satisfaction, role conflict and ambiguity.^31^

The provision of psychological support to student nurses was also mentioned. A dedicated service for them to deal with their day to day to psychological trauma of death and suffering has to be in place.^32^ In the recent COVID pandemic more than one suicide could have been avoided.^33^ Some valid suggestions on the day to day running of the wards were provided to the administrators who welcomed and tried to address them with fruitful outcomes.

It is surprising how these young student nurses provided such astute observations which were equal to some high powered and expensive studies. Today’s students are wise and observant, and are not afraid to participate in improvement of the patient care. Information from such questionnaires can provide timely feedback and enable administrators to identify and correct organizational factors.^34^ ***If only we would listen to them actively*.**

### Limitations of the Study

This being first study of its type, it has some limitations. The study was conducted in one nursing institute and therefore, the findings can only be used to this specific participant group.

## CONCLUSION

Doctors need to improve their counseling skills and should talk more to the patients and their relatives. They should also acknowledge the nursing students and take them along to improve the teamwork. Belittling them in front of others on the ward harms their self-efficacy and capacity to function maximally. Simple corrections like punctuality, ownership of their patients and improvement of systems can enhance the quality of care.

### Author’s Contributions:

**SAS:** Conception and design, entering, analysis and interpretation of data, drafting the article, literature search, referencing and revising critically.

**HZ:** Facilitating administration of proforma, collection of data, revision of draft, literature search.

**JA:** Improvement of proforma, analysis and interpretation of data, literature search, intellectual input and revision of draft.

**RQ:** Development of proforma, analysis of results, intellectual input, revision of draft.

**SN:** Analysis of results, corrections & revision of draft, intellectual input.

**CAS:** Conception and design, administering the survey, analysis and acquisition of data, interpretation of data, revising the draft, final approval, agreement to be accountable for all aspects of the work, corresponding author.
